# Supplementary material for: Seroprevalence of Hantavirus in Forestry Workers, Northern France, 2019–2020
Source: Viruses. 2023 Jan 25;15(2):338. doi: 10.3390/v15020338 (PMC9962707; doi:10.3390/v15020338)
Supplement: Supplementary file 1 [file viruses-15-00338-s001.zip › viruses-2134376-supplementary.pdf]

Supplementary material

Seroprevalence of Hantavirus in Forestry Workers, Northern France, 2019–2020

Catarina Krug [1,2], Emma Rigaud [3], Dieyenaba Siby-Diakite [4], Laetitia Bénézet [1], Pavlos Papadopoulos [4], Henriette de Valk [1], Gaëtan Deffontaines [3], Alexandra Septfons [1,†] and Jean-Marc Reynes [4,*,†]

[1] Santé publique France, 94410 Saint-Maurice, France
[2] European Centre for Disease Prevention and Control (ECDC), 169 73 Solna, Sweden
[3] Caisse Centrale de la Mutualité Sociale Agricole, 93000 Bobigny, France
[4] Institut Pasteur, Université Paris Cité, 75015 Paris, France
* Correspondence: jean-marc.reynes@pasteur.fr; Tel.: +33-1-40-61-38-08
† These authors contributed equally to this work.

Appendix A

Using a 5% alpha, and assuming that the hantavirus seroprevalence among forestry workers lies between 0.5 and 10%, we will be able to estimate overall seroprevalence with a precision (i.e. margin of error) between 0.3% and 1.4% (Table S1). Clustering of observations by region (i.e. dependency of data between individuals working at the same region.) was not considered for these calculations, and tests were assumed to have perfect sensitivity and specificity; therefore, the precision presented is likely to be slightly optimistic.

Table S1. Precision of hantavirus seroprevalence estimates, assuming a sample size of approximately 1,781 individuals and a true seroprevalence lying between 0.5 and 10%.

Sample size	Prevalence (%)	Precision (%)
1,781	0.5	0.327
1,781	1	0.460
1,781	2	0.650
1,781	3	0.790
1,781	5	1.010
1,781	10	1.390
